# Publications Among Pain Medicine Fellowship-Trained American Board of Anesthesiology Diplomates

**DOI:** 10.7759/cureus.61821

**Published:** 2024-06-06

**Authors:** James H Jones, Neal Fleming

**Affiliations:** 1 Anesthesiology, University of North Carolina-Chapel Hill, Chapel Hill, USA; 2 Anesthesiology and Pain Medicine, University of California (UC) Davis Medical Center, Sacramento, USA

**Keywords:** anesthesiology, research productivity, fellowship, pain medicine, graduate medical education

## Abstract

American Board of Anesthesiology (ABA) diplomates who pursue clinical fellowship training in pain medicine may be better suited to lead scholarly projects and serve as first authors of publications in peer-reviewed journals given their additional training and clinical expertise. The primary aim of this study was to determine whether ABA certification in pain medicine is associated with a greater number of peer-reviewed publications. The secondary aim included assessments of whether pain medicine fellowship training is associated with a higher publication rate (publications per year) or publication in a larger number of peer-reviewed journals. A literature search was conducted in December 2023 using the Scopus database for publications related to anesthesiology and pain medicine in the United States between 2013 and 2023. First authors identified through the search were then individually searched within the ABA physician directory. The following data were collected: author name and identification number, year of publication, publication type (article or review), year of primary anesthesiology certification, and year of fellowship, if applicable. This study identified 9,612 publications and 6,924 unique first authors. Pain medicine fellowship training was associated with a statistically significant increase (p-value < 0.001) in the number of publications (0.546; 95% confidence interval {CI}, 0.386-0.707), publications per year (0.140; 95% CI, 0.121-0.159), and publication in a larger number of peer-reviewed journals (0.256; 95% CI, 0.182-0.330) in regression models adjusted for the number of years from certification. This query of the Scopus database and ABA physician directory indicates that pain medicine fellowship training is associated with statistically significant increases in research productivity, as defined by the number of publications, publications per year, or the number of publications in peer-reviewed journals. However, these increases in research output would not lead to a marked increase in scholarship productivity to justify pursuing a fellowship for this purpose.

## Introduction and background

American Board of Anesthesiology (ABA) diplomates who pursue clinical fellowship training in pain medicine may be better suited to lead scholarly projects and serve as first authors in peer-reviewed journals given their advanced clinical training and clinical expertise. Academic medical centers and professional designations frequently consider scholarly efforts and productivity when evaluating faculty [1]. Scholarly activity is also a required component of training for all pain medicine fellows [2]. The Accreditation Council for Graduate Medical Education (ACGME) Program Requirements for Graduate Education in Pain Medicine states, "All fellows must complete a scholarly project. The results of such projects must be disseminated through a variety of means, including publication or presentation at local, regional, national, or international meetings" [2]. The limited research activity of anesthesiologists has been well-documented and threatens the future of academic anesthesiology [3].

Prior researchers have investigated the impact of fellowship training on scholarly production in anesthesiology and other medical specialties [4-6] but with methods that limit the generalizability of the results, such as restricting searches to randomly selected anesthesiology departments or only new ABA diplomates [7,8] and the use of summary measures such as the h-index. The primary aim of this study was to determine whether ABA certification in pain medicine is associated with a greater number of peer-reviewed publications. The secondary aim included assessments of whether pain medicine fellowship training is associated with a higher publication rate (publications per year) or publication in a larger number of peer-reviewed journals.

## Review

Methods

Search Strategy

The Scopus database was searched in December 2023 for articles with the following query: "anesthesia" OR "anesthesiology" OR "anaesthesia" OR "anaesthetic" OR "anesthetic" OR "pain" OR "pain medicine" OR "analgesia." The first author's name was recorded for each article. The ABA physician directory was then individually searched for each author's name obtained from the Scopus database search.

Eligibility and Exclusion Criteria

Open access journal articles and reviews written in English in the United States between 2013 and 2023 in the "medicine" subject area were included for review. Additional keywords, funding sponsor, and affiliation were not specified to maximize the included publications. The following journals were included in the selection of relevant studies: Anesthesia & Analgesia, Anesthesia Progress, Anesthesiology, BMC Anesthesiology, British Journal of Anaesthesia, Canadian Journal of Anesthesia, The Clinical Journal of Pain, Cochrane Database of Systematic Reviews, European Journal of Pain (United Kingdom), Frontiers in Pain Research, The Journal of the American Medical Association (JAMA), JAMA Network Open, Journal of Cardiothoracic and Vascular Anesthesia, The Journal of Headache and Pain, The Journal of Pain, Journal of Pain and Symptom Management, Journal of Pain Research, The Lancet, Neuromodulation, The New England Journal of Medicine, Paediatric Anaesthesia, Pain, Pain Medicine (United States), Pain Reports, Pain and Therapy, and Regional Anesthesia & Pain Medicine.

Study Selection and Data Collection

All searches were performed on publicly available databases, thus precluding the need for institutional review board (IRB) review and approval. The corresponding author independently reviewed and extracted the following data from each eligible article identified in the Scopus database: first author's full name, first author's open researcher and contributor ID (ORCID) number, year published, journal title, and document type (review or original research article). The following data were then collected from the ABA physician directory: primary anesthesiology or fellowship training certification(s) and year certification(s) issued, if applicable. Authors who were not so identified in the ABA physician directory were assumed not to have completed a chronic pain medicine fellowship.

Names with initials from the Scopus database, such as J. Doe, were only considered present in the ABA directory if only one name matched the available name components (such as John Doe). However, if there were multiple first names with the same first letter (such as John Doe and Jason Doe) in the ABA directory, then it was assumed that the author (J. Doe) did not have a clinical fellowship. The reverse situation, where only initials are present in the ABA directory but full names are present in the Scopus database, was handled similarly.

Definitions

The following variables were defined a priori.

"Years from certification": This is the difference in the number of years between the article publication date and initial fellowship certification.

Clinical fellowship: This is present if author and certification are identified in the ABA physician directory.

Professionally significant impact: Linear regression model coefficients for the number of publications, publication rate, or publications in different journals greater than or equal to one (translating to one publication or, for publication rate, one publication per year over the 10-year search window) with a p-value of <0.05 were considered significant within the context of a professional career. This definition was decided upon, a priori, in an effort to provide some context to decide what is a meaningful result and what is likely not a meaningful result.

Publication rate: This is the quotient of the number of publications and the lesser of "years since certification" or 10 (because only publications between 2013 and 2023, a total of 10 years, were included in this study). This timeframe was chosen to capture the current trends in research output among fellowship-trained pain physicians.

Statistical Analyses

The primary outcome measure for this study was the number of publications from ABA diplomates with pain medicine fellowship training compared to those without a pain fellowship. A multivariable linear regression model was chosen to statistically analyze the primary outcome, adjusted for "years from certification." The secondary outcome measures for this study were the publication rate and number of journals with publications from ABA diplomates with pain fellowship training compared to those without a pain fellowship. A multivariable linear regression model was chosen to statistically analyze the secondary outcome, adjusted for "years from certification." Authors who were not so identified in the ABA directory were assumed not to have completed a clinical fellowship and were therefore analyzed in the same group as diplomates identified in the ABA directory but without a clinical fellowship. All analyses were performed with Stata software, release 17 (StataCorp LLC, College Station, TX).

Results

Population Selection

**Figure 1:**
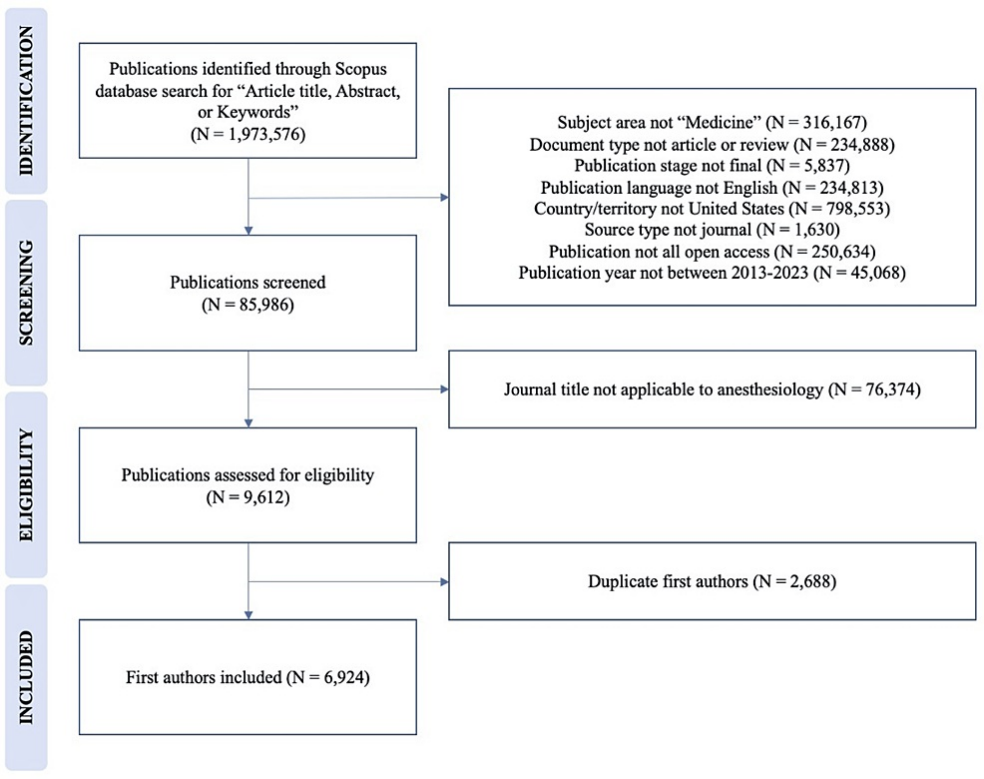
Identification and Screening of Publications in the Scopus Database

The Scopus database search initially identified 1,973,576 publications. A total of 85,986 publications were screened after exclusion based on the following filters: subject area (medicine), document type (article or review), publication stage (final), language (English), country/territory (United States), source type (journal), source title, access designation ("all open"), and date range (2013-2023). In total, 9,612 publications were eligible for study inclusion. From these publications, 6,924 distinct first authors were identified and served as the population subsequently queried in the ABA physician directory. Figure 1 displays the identification and screening of publications from the Scopus database.

Characteristics of Included First Authors and Journals

Most (90.01%) of the identified first authors (n = 6,232) did not appear in the ABA physician directory or had no fellowship training designation, 290 (4.19%) first authors had a pain medicine fellowship, and 402 (5.81%) first authors had fellowship training in a specialty other than pain medicine. Among all publications identified in the Scopus database, the most common journal topics were pain (55.55%), followed by anesthesiology (28.47%), medicine (9.65%), and reviews (3.55%). Of the 290 physicians with pain fellowship training, 10 of them also had a fellowship in hospice and palliative medicine. Unclarity regarding an author's clinical fellowship status due to the way in which their names were presented in the ABA directory and Scopus database occurred in 179 of 9,611 total cases (1.86%). Descriptive statistics of the data are shown in Table 1. Kruskal-Wallis tests demonstrated significant differences in the average number of publications and the number of publications per year, while Pearson's test demonstrated significant differences in the number of publications in different peer-reviewed journals among physicians with pain medicine fellowship compared to those without fellowship or other fellowship training.

**Table 1:** Descriptive Statistics of the Included First Authors Data are presented as median (interquartile range) for continuous variables or percent for the number of journals > 1

Characteristics	All First Authors (N = 6,924)	No Fellowship (N = 6,232)	Pain Medicine Fellowship (N = 290)	Fellowship Not Pain (N = 402)	P-value
Number of Publications	1 (1-1)	1 (1-1)	1 (1-2)	1 (1-2)	<0.001
Publications/Year	0.1 (0.1-0.111)	0.1 (0.1-0.1)	0.167 (0.1-0.333)	0.125 (0.1-0.25)	<0.001
Number of Journals > 1	13%	12%	26%	18%	<0.001

Primary Outcome: Number of Publications

A multivariable linear regression model adjusted for "years from certification" demonstrated that the difference in the number of publications between diplomates with and without pain medicine fellowship training is statistically significant (p-value < 0.001). However, put another way, in comparing two diplomates with the same number of years from initial certification, the diplomate with pain medicine fellowship training will produce an average of 0.546 publications more over a 10-year period than the diplomate without a pain fellowship. In contrast, when comparing two diplomates with the same number of years from initial certification, the diplomate with fellowship training in a subspecialty other than pain medicine will produce an average of 0.110 publications more over a 10-year period than the diplomate without fellowship, but this value is not statistically significant (95% confidence interval {CI}, -0.0198-0.240; p-value, 0.097).

*Secondary Outcomes: Publication Rate and Journal Number*

Multivariable linear regression models adjusted for "years from certification" demonstrated that pain medicine fellowship training is associated with a statistically significant (p-value < 0.001) increase in the number of publications per year (0.140 with 95% CI: 0.121-0.159) and the number of publications in different journals (0.256 with 95% CI: 0.182-0.330). Comparing two diplomates with the same number of years from initial certification, the diplomate with pain medicine fellowship training produces, on average, 0.140 publications per year more and publishes their work in 0.256 more journals, on average, compared to the diplomate without a pain fellowship over a 10-year period. Table 2 displays the results from regression models investigating the association of pain medicine fellowship certification on publication number, publication rate, and the number of journals.

**Table 2:** Association of Pain Fellowship on the Number of Publications, Publication Rate, and the Number of Journals *Categorical variable with no fellowship as a comparison group **Fellowship other than pain medicine N, number; Coeff, beta coefficient; CI, confidence interval

Variable	Publication Number (N = 6,730)			Publication Rate (N = 6,730)			Journal Number (N = 6,730)		
	Coeff	95% CI	P-value	Coeff	95% CI	P-value	Coeff	95% CI	P-value
Fellowship*									
Pain Medicine	0.546	0.386-0.707	<0.001	0.140	0.121-0.159	<0.001	0.256	0.182-0.330	<0.001
Others**	0.110	-0.0198-0.240	0.097	0.0547	0.0391-0.703	<0.001	0.0986	0.0386-0.158	0.001
Years From Certification	0.0211	0.0161-0.0262	<0.001	0.00119	0.000588-0.00179	<0.001	0.00608	0.00376-0.00839	<0.001

Discussion

ABA diplomates with advanced clinical training in pain medicine publish more manuscripts at a faster rate and in more journals compared to diplomates without this clinical specialization. This increase in research productivity is statistically significant in all models adjusted for years from certification. However, none of these differences would be considered significant within the context of an academic career when interpreting these results based on our definition of a "professionally significant impact" as defined in the "Methods" subsection.

Surgical specialties have investigated whether research productivity is enhanced with specialty training certification with disparate results [4-6]. Publication rates from urology faculty did not appear to be significantly associated with fellowship certification in a study that queried the Scopus database and measured research productivity with the h-index (18.1 ± 0.95 and 14.62 ± 0.80 for urologic oncologists and non-fellowship-trained urologists, respectively) [5]. Conversely, ophthalmologists with fellowship training, particularly those with training in vitreoretinal disease or ophthalmic pathology, exhibited greater research productivity, as measured by the h-index, compared to non-fellowship-trained ophthalmologists [6]. Researchers investigating scholarly production among spine fellowship programs found greater productivity using both absolute numbers of publications and h-indices for faculty of programs with academic affiliations and programs with a greater number of fellows [4].

The impact of clinical fellowship training on research productivity for subspecialty-trained anesthesiologists has been previously investigated, albeit with different methods from this study. Eloy et al. defined research productivity with the h-index and reviewed faculty from just 20 randomly selected anesthesiology departments [7]. However, their results did not demonstrate a statistically significant difference in research productivity between faculty with fellowships compared to those without (2.88 versus 2.98, respectively) [7]. Ford et al. found a doubling in the mean number of publications (0.31-0.79) per diplomate from 2006 to 2016 [8]. Their search, however, was limited to new ABA diplomates. Pagel and Hudetz used the h-index as a marker of scholarly efforts in a single-point-in-time comparison of faculty in 30 randomly selected departments with adult cardiothoracic fellowship training programs either with or without ACGME accreditation [9]. They found that academic rank and ACGME accreditation positively influenced h-index values. O'Leary and Crawford also used the h-index to characterize the academic productivity of anesthesiologists in all university-affiliated departments of pediatric anesthesia in Canada [10]. The authors found that the h-index increased in association with academic seniority.

The h-index attempts to quantify the impact of an author's manuscripts by measuring its citations by other published manuscripts, such that an h-index of 5 indicates that the author has five publications with at least five citations [11]. Consequently, the h-index is a more appropriate marker for a manuscript's impact and not necessarily a robust characterization of the efforts of individual authors with varying levels of contribution, different areas of clinical expertise, and disparate research opportunities [12]. Furthermore, the h-index characterizes scholarly production over the course of an academic career and may not be adequately controlled in regression models that focus on a restricted period of time (10 years in this manuscript). Additionally, authors who publish articles that are not frequently cited will have a lower h-index and thus fail to capture research productivity. The current manuscript only considers the first authors, who likely provided the most meaningful contributions to the publications, which is not apparent in analyzing an author's h-index. Furthermore, we did not limit our study population to a limited set of anesthesiology departments, thus making our results more generalizable to ABA diplomates.

To this point, the medical literature has not investigated the association between clinical fellowship training in pain medicine and research productivity, thus justifying the importance of this research. Physicians have been board-certified in pain medicine since 1993 with programs first becoming ACGME-accredited in 1992 [13]. This timeframe is more recent than critical care (established in 1985) but older than adult cardiac anesthesiology (accreditation in 2006), obstetric anesthesiology (2012), pediatric anesthesiology (accreditation in 1997 with first certification examination in 2013), and regional anesthesiology and acute pain medicine (2016) [14-17]. According to the ACGME Data Resource Book, there are 114 accredited programs for pain medicine [18]. The anesthesiology fellowship with the next most programs is adult cardiothoracic medicine (74 programs), followed by critical care (64 programs), pediatric (61 programs), obstetric (41 programs), and regional anesthesia and acute pain medicine (40 programs) [18].

There is a broad spectrum of publication options in peer-reviewed journals for anesthesiologists with fellowship training in pain medicine. Thirteen of the journal titles included in this review include the word "analgesia" or "pain." Although there appears to be some potential advantage for diplomates with fellowship training in pain medicine to lead more productive research careers, the current manuscript did not find substantial practical evidence to support this potential advantage.

Limitations

The ABA physician directory may not serve as a definitive dataset for statistical analyses. All authors with advanced expertise in clinical areas may not be appropriately identified in the ABA directory. In fact, diplomates known by the authors of this manuscript as having clinical fellowships were not listed in the ABA physician directory as such. The reasons for this discrepancy in diplomate status and clinical specialization may include graduation from a nonaccredited program, incomplete ABA directory data, and failure to complete all requirements for certification. Data in the ABA physician directory come from diplomates, training programs, and the results from certification examinations. The way names are presented in the directory is, therefore, at the discretion of each diplomate, thus serving as a major limitation in matching names from the Scopus database with those in the ABA physician directory. The authors included in this manuscript's dataset may share the same name as an ABA diplomate and be inappropriately identified as having a clinical fellowship. Conversely, and more likely, our dataset may miss fellowship-trained authors. Authors who only report initials for their first or last names may have been excluded from the dataset due to multiple diplomates with the same initials. Without more specific identification than an author's name (such as social security number and birth date), a more accurate dataset is not feasible.

Because pain medicine is an interdisciplinary specialty, physicians may be listed in the American Board of Physical Medicine and Rehabilitation (ABPMR) or American Board of Psychiatry and Neurology (ABPN) directories as their primary specialty as opposed to the ABA physician directory; as such, the research efforts of neurologists or physiatrists would be less likely to have been captured in the initial data search, which focused on anesthesiology as the primary specialty. Additionally, this manuscript only considered the first authors and did not investigate the additional authors for each manuscript. Lastly, scholarly production is not necessarily synonymous with peer-reviewed publications and should also consider conference presentations, grant awards, book chapters, and educational efforts, among other academic activities.

Despite statistical significance, none of our results demonstrate meaningful increases in research productivity with pain medicine fellowship training because the increase in the number of publications, publications per year and the number of peer-reviewed journals is all less than one. Additional research is needed to identify variables that may have a greater impact on research productivity in anesthesiology diplomates, such as department funding, academic rank, mentorship programs, or advanced research degrees (Master of Science, Doctor of Philosophy, etc.). The regression models used in this manuscript do not account for all variables associated with research production. Alternative statistical models might account for random yet unidentified variables, such as a linear mixed-effect model with random intercepts for author ID. We considered using a mixed-effect model instead of a regression model, but few authors with pain fellowship (n = 28, 9.66%) were responsible for more than two publications, thus diminishing the value of analyses that consider the random effects from individual authors. Research productivity is driven by innumerable personal factors and institutional resources that are difficult to quantify and cannot be reasonably included in statistical models.

## Conclusions

This query of the Scopus database and ABA physician directory indicates that pain medicine fellowship training is not associated with meaningful increases in peer-reviewed research publication, as defined by the number of publications, publication rate, or publications in different journals.
